# Personal

**Published:** 1907-03

**Authors:** 


### PERSONAL.

Dr. John Young Brown, superintendent and surgeon in charge of the city hospital at Saint Louis, was recently appointed chief surgeon to St. John's Hospital in that city. Dr. Brown was also unanimously elected professor of clinical surgery by the faculty of Saint Louis University, to succeed Dr. A. V. L. Brokaw, deceased. During the four or five years that Dr. Brown has been in charge of the city hospital he has transformed it from a condition almost bordering on a pest house to one of the largest and best equipped hospitals in this country. He has nearly or quite thirty assistants under him, and has performed all the operations known to surgery with results that are second to none. Dr. Brown is a native of Kentucky, son of the late Governor John Young Brown, and by birth, education, and training is everyway fitted for the new positions which he is about to undertake and which will soon necessitate the yielding of his present charge to enter upon private surgical practice.

Dr. Charles Haase, a graduate of the University of Buffalo, 1902, and more recently assistant surgeon at the State Soldiers' and Sailors' Home at Bath, has lately sailed for Europe for recreation and study. It is his announced intention to pay a three months' visit to the Mediterranean region and afterward engage in a year's medical study in Vienna.

William Nottingham, of Syracuse, was reelected a regent of the University of the State of New York by both houses of the legislature, February 12, for the full term of eleven years, beginning April 1, 1907.

Dr. J. E. Walker, superintendent of the Steuben Sanitarium, Hornell, N. Y.. who has been spending some months in the south of Europe, has returned to his home and resumed his professional work.

Dr. R. Park, of Buffalo, has been appointed a visitor to attend the West Point graduating exercises in June, 1907.

